# Mechanistic Insight into Long Noncoding RNAs and the Placenta

**DOI:** 10.3390/ijms18071371

**Published:** 2017-06-27

**Authors:** Dale McAninch, Claire T. Roberts, Tina Bianco-Miotto

**Affiliations:** 1 Adelaide Medical School & Robinson Research Institute, University of Adelaide, Adelaide, SA 5005, Australia; dale.mcaninch@adelaide.edu.au (D.M.); claire.roberts@adelaide.edu.au (C.T.R.); 2 School of Agriculture, Food and Wine, Waite Research Institute & Robinson Research Institute, University of Adelaide, Adelaide, SA 5005, Australia

**Keywords:** placenta, lncRNA, long non-coding RNA, lincRNA, pregnancy, ncRNA, preeclampsia

## Abstract

Long non-coding RNAs (lncRNAs) are classified as RNAs greater than 200 nucleotides in length that do not produce a protein product. lncRNAs are expressed with cellular and temporal specificity and have been shown to play a role in many cellular events, including the regulation of gene expression, post-transcriptional modifications and epigenetic modifications. Since lncRNAs were first discovered, there has been increasing evidence that they play important roles in the development and function of most organs, including the placenta. The placenta is an essential transient organ that facilitates communication and nutrient exchange between the mother and foetus. The placenta is of foetal origin and begins to form shortly after the embryo implants into the uterine wall. The placenta relies heavily on the successful differentiation and function of trophoblast cells, including invasion as well as the formation of the maternal/foetal interface. Here, we review the current literature surrounding the involvement of lncRNAs in the development and function of trophoblasts and the human placenta.

## 1. Introduction

More than 75% of the human genome is actively transcribed into non-coding RNAs (ncRNA), which is a stark contrast to the 1–2% of the genome that is protein coding [1,2]. These ncRNAs are comprised of both small RNAs, including but not limited to micro RNA (miRNA), piwi-interacting RNA (piRNA), small interfering RNA (siRNA), small nucleolar RNA (snoRNA), and transfer RNA (tRNA) [3]. A large number of ncRNAs are >200 nucleotides in length and are termed long non-coding RNAs (lncRNA) [3]. These are generally poorly conserved between species, for example, only 14% of mouse lncRNAs have a human orthologue [4,5]. Since the introduction of next generation sequencing there has been a steady increase in the number of lncRNAs identified. Currently it is estimated that there are more lncRNAs than protein coding genes in the genome [2].

ncRNAs have increasingly appreciated roles in all stages of development, with each type of ncRNA having distinct functions and mechanisms of action. miRNAs act by binding complimentary RNA sequences where they recruit proteins that can destabilise RNA or impair translation leading to a reduction in target protein levels [6,7]. lncRNAs have a diverse range of molecular functions. They act as scaffolds, signals, and antisense decoys, and engage in transcriptional interference. It is not uncommon for a single lncRNA to have multiple functions [8,9]. Scaffold lncRNAs bind proteins and other RNAs to form larger functional complexes, such as the telomerase RNA complex required for telomere repeat synthesis [10] or the polycomb repressor complex involved in histone modifications [11]. lncRNAs can act as signals of particular developmental stages, transcriptional regulation or specific locations. The transcription of some lncRNAs can indicate the silencing of co-located protein coding genes [12], or act as markers of upstream transcriptional events. Decoy lncRNAs can act by binding a target protein, preventing the protein from performing its normal function [13,14,15] or by binding and sequestering small regulatory RNAs including miRNAs [16]. Although the number of identified lncRNAs continues to grow, the function and biological significance of the majority of lncRNAs remain unknown. lncRNAs play an important role in regulating gene expression in utero, and likely strongly influence postnatal development and future health.

## 2. The Placenta

The placenta is one of the products of conception and, thus, is genetically identical to the foetus. Correct placental development and function is critical for both maternal and foetal health [17]. The placenta is central to the cross talk between mother and foetus. It provides many functions beyond the transfer of oxygen, nutrients and water to the foetus and elimination of wastes from it. These include secretion of hormones into the maternal circulation that orchestrate maternal adaptation to pregnancy and transfer of maternal antibodies to the foetus to provide passive immunity to pathogens the newborn is likely to encounter [17]. Defects in placental differentiation and function have been associated with pregnancy complications.

There are some published reports on placental lncRNAs in pregnancy complications assessed after delivery but there is a paucity of literature on the role of lncRNAs in normal placental development.

## 3. The Role of lncRNAs in Pregnancy Complications

Abnormal placental differentiation has been implicated in a number of pregnancy complications such as miscarriage, preeclampsia and intrauterine growth restriction [18,19]. Pregnancy complications can have a lifelong influence on not only the mother’s health, but also that of her offspring. The long-term impact on offspring health results from foetal programming which heightens risk of developing non-communicable diseases later in life [20]. Identifying how these pregnancy complications arise is a critical step for prevention. Gaining a greater understanding of the genetic and molecular pathways involved in disease pathogenesis is essential and may provide future targets for therapeutic action. An advantage to targeting lncRNAs over messenger RNAs (mRNA) is that targeting a single lncRNA has the potential to affect multiple downstream pathways and therefore amplify the effect of a single target. Targeting multiple pathways may also lead to unwanted side effects, so the functions of lncRNAs should be completely characterised before they are used for therapeutic targets. There are currently many different methods being developed to silence lncRNAs in cancer, including siRNAs, antisense transcripts and aptamers, that could potentially be adapted for use in treating pregnancy complications [21].

**Figure 1:**
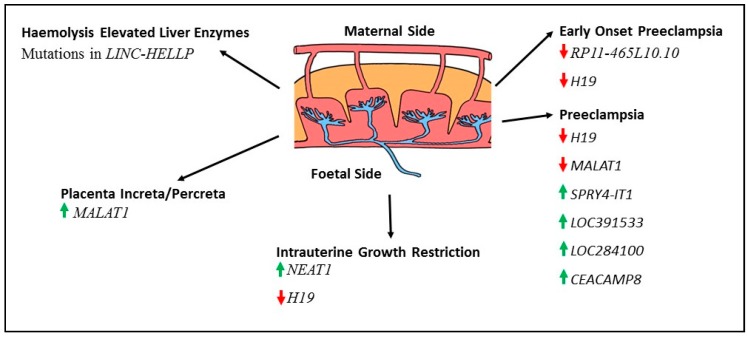
Summary of lncRNAs reported as altered in pregnancy complications. Green arrows indicate lncRNAs that are increased in pregnancy complications when compared to control samples and red arrows indicated those that are decreased in expression.

Table 1 and Figure 1 summarizes the lncRNAs linked to pregnancy complications. Additional studies are required to confirm if the changes in expression are the cause or the consequence of these complications.

### 3.1. Miscarriage

Miscarriage is defined as pregnancy loss that occurs before 20 weeks of gestation. Miscarriage during the first trimester affects up to 15% of clinically recognised pregnancies and up to 50% of recurrent pregnancy losses have no identifiable cause [34]. Up to 50% of first trimester miscarriage is associated with aneuploidy and chromosome translocations while other factors include maternal age, obesity, thrombophilia, smoking, alcohol use, endocrine and immune parameters [35]. To date there have only been two studies identifying a link between altered lncRNA expression and miscarriage. The first study used microarray analysis to compare the expression of lncRNAs by embryonic sac/chorionic villi in miscarriage with those in gestation matched pregnancies with elective induced abortion and identified a large number of differentially expressed lncRNAs [36]. A number of the regulatory pathways represented by these lncRNAs are related to infection and inflammation (although the majority of miscarriages show no sign of infection), metabolism, signalling, and transcription regulation [36]. In a recent study, also using the same microarray approach, 1449 differentially expressed lncRNAs were identified from chorionic villi tissue from recurrent miscarriages (women who had suffered three or more miscarriages before 12 weeks of gestation) compared to normal pregnancy [37]. Some of the pathways represented by these differentially expressed lncRNAs included immunity, steroid hormone biosynthesis and transforming growth factor beta (TGF-β) signalling pathway [37].

### 3.2. Preeclampsia

Preeclampsia (PE) is a pregnancy complication diagnosed by new onset hypertension after 20 weeks of gestation with either proteinuria and/or other maternal organ dysfunction and/or foetal growth restriction [38]. It is characterised by systemic maternal oxidative stress and endothelial dysfunction [39,40]. PE occurs in 3–5% of pregnancies worldwide and is a major cause of maternal and perinatal mortality [41,42]. Microarray analysis (33,045 total probes for lncRNAs) comparing placentas from PE and control pregnancies showed 738 (259 up and 479 down) lncRNAs were differentially expressed [22]. *LOC391533*, *LOC284100* and *CEACAMP8* are three lncRNAs found to be more highly expressed in placenta from PE and have been validated by quantitative polymerase chain reaction (qPCR) comparing placentas from 40 PE and 40 control pregnancies. A co-expression analysis of lncRNA and mRNA differential expression identified significant enrichment for pathways involving lipid metabolism and type 2 immune response, consistent with the concept that PE has a disrupted metabolic and immune state compared to uncomplicated pregnancies [22].

During normal placentation, extravillous cytotrophoblast cells colonise and migrate within the spiral arteries in a retrograde direction transforming their structure to form large compliant vessels lacking their smooth muscle layer [43]. This invasion and remodelling is required for successful pregnancy, whereas in PE this arterial transformation fails or is impaired. A comparison of placentas from 25 control and 25 PE pregnancies found a 2.8-fold increase in the expression of the lncRNA *SPRY4-IT1* (708 bp) [23]. *SPRY4-IT1* has been linked to the formation of endothelial cell-like tubular networks (as discussed below), an important angiogenic process, and may implicate this lncRNA in spiral artery remodelling, in particular in placentas from PE pregnancies where it has higher expression compared to in uncomplicated pregnancies.

A lncRNA microarray comparison of placentas from six early onset PE (EOPE) pregnancies and six preterm deliveries as gestational age matched controls found 15,646 lncRNAs were up regulated and 13,178 were down regulated in EOPE [25]. As the function of the majority of lncRNAs remains largely unknown, standard gene ontology (GO) pathway analysis cannot be performed. In an attempt to assign a function to the differentially expressed lncRNAs, the neighbouring protein coding genes were interrogated by GO which revealed that pathways relating to cell migration and motility were significantly enriched [25]. The lncRNA *RP11-465L10.10* was found to be downregulated in placentas from cases of EOPE, along with its target gene *MMP9* [25]. Studies of *MMP9* have linked its expression to PE and trophoblast invasion in women [44,45]. It is important to note that approximately 80% of the lncRNA probes on the microarray were identified as being differentially expressed, which has implications for interpretation of the analysis. Since preterm birth is itself a pregnancy complication, using placentas from preterm pregnancies may not constitute an ideal control so these results should be viewed with caution.

Altered *MALAT1* expression has been found in the placenta of two different pregnancy complications, PE and placenta increta/percreta. Placentas from PE pregnancies show significantly lower expression of *MALAT1* compared to control placentas [24], while placentas from women with placenta increta/percreta show significantly higher expression of *MALAT1* [29]. *MALAT1* expression increases invasion and migration in trophoblast cell lines, as discussed below. The loss of expression of *MALAT1* in PE placentas may lead to a reduction in the invasive properties of trophoblast cells resulting in PE pathogenesis. Conversely, the overexpression of *MALAT1* observed in placenta increta/percreta may lead to an overly invasive trophoblast phenotype. It should be noted that altered *MALAT1* expression has only been shown after trophoblast invasion has occurred; as such, further evidence is required to determine if altered *MALAT1* is a cause or merely a marker of altered trophoblast invasion in these pregnancy complications.

Haemolysis, elevated liver enzymes, low platelet count (HELLP) syndrome is considered a severe form of preeclampsia [46,47,48]. HELLP symptoms usually present later in pregnancy, however first trimester placentas from women with HELLP show a reduction in trophoblast invasion and impaired spiral artery remodelling [49]. *LINC-HELLP* is a 205-kb lncRNA located within a genomic region linked to familial HELLP syndrome in Dutch women [27]. *LINC-HELLP* is found within both the nucleus and cytoplasm of first trimester extravillous cytotrophoblast cells [28]. Single and compound mutagenesis studies of *LINC-HELLP* have demonstrated that this lncRNA plays an important role in trophoblast proliferation and invasion [28]. Compound mutations found within families that have HELLP syndrome such as the HAPLO215Rev + HAPLO378-M1 compound mutation leads to a significant increase in trophoblast proliferation and a significant reduction in invasion [28]. These changes to proliferation are consistent with a cell cycle exit phenotype. It was also observed that mutations in *LINC-HELLP* can reduce the differentiation of extravillous trophoblast cells [28]. *LINC-HELLP* has been shown to interact with ribosomal proteins RPS6 and RPL7 in SGPHL-5 cells (an extra villous trophoblast like cell line) [28]. While lncRNAs are not usually associated with the ribosome, as they are not translated, this interaction between lncRNAs and the ribosome has been observed for other lncRNAs and may be linked to lncRNA degradation [50]. It is still unclear as to whether this interaction is a specific function of particular lncRNAs or if it is a coincidental interaction due to the RNA binding nature of the ribosome. *LINC-HELLP* knockdown followed by RNA-Seq shows a massive reduction in gene expression [28], which may link this lncRNA to transcriptional and/or posttranscriptional regulation via the ribosome and RNA splicing machinery.

### 3.3. Intrauterine Growth Restriction

Intrauterine growth restriction (IUGR) is defined as impaired foetal growth where the foetus does not achieve its expected growth potential given its race, sex and gestation. IUGR is well known to increase the risk for perinatal morbidity and mortality [17]. Undetected IUGR is a significant antecedent to stillbirth [51]. IUGR is also known to be a significant risk factor for adult onset non-communicable diseases including cardiovascular disease and Type 2 diabetes and as the placenta mediates nutrient transport to the foetus in response to foetal demand and maternal capacity, it is key to foetal programming [52].

A complimentary DNA (cDNA) subtraction assay comparing placentas from 12 IUGR and 12 control pregnancies revealed overexpression of the lncRNA *NEAT1* in IUGR [53]. *NEAT1* expression was observed by in situ hybridisation and was localised exclusively within villous trophoblast cells of the placenta. In addition, more cells express *NEAT1* in IUGR villous trophoblast cells compared with control placenta. There are two isoforms of the lncRNA *NEAT1*, *NEAT1_1* and *NEAT1_2. NEAT1_2* is required for the formation of paraspeckles, while *NEAT1_1* has been shown to increase the number of paraspeckles found in a nucleus when overexpressed [30]. *NEAT1* functions by retaining hyper-edited mRNAs in the nucleus. Nuclear paraspeckles are sub nuclear regions where transcription and pre-mRNA transcript processing occurs [54]. Interestingly, paraspeckles have been linked to cell growth and differentiation [55], and the disruption of paraspeckles is often found in disease states, such as acute promyelocytic leukaemia [56].

## 4. *H19*

*H19* was one of the first lncRNAs to be discovered [57,58], long before the introduction of next generation sequencing; consequently, it is the most highly studied lncRNA. *H19* is located within a large imprinted domain on chromosome 11, 130 kb downstream of *IGF2* [59,60]. *H19* and *IGF2* are reciprocally imprinted that is, for *H19* only the paternal allele is expressed, while for *IGF2*, only the maternal allele is expressed [60]. *H19* expression can be regulated by *PLAGL1*, a zinc finger containing transcription factor, in the human placenta [61]. It has been proposed that due to the high level of sequence conservation, the secondary structure of *H19* is very important for its function [62]. Two major functions have been described for *H19*, specifically a modulator for binding small RNAs and proteins [63], and as a source of the miRNA mir-675 [64], this function of *H19* has been recently reviewed [65].

DNA methylation of gene regulatory regions typically leads to a reduction in gene expression (however, this is not always the case [66,67]). Additionally, DNA methylation plays a major role in genomic imprinting, leading to parent-of-origin specific allele expression. *H19* has variable levels of biallelic expression in the placenta (reports suggest between 9% and 25% expression occurs from the imprinted allele) until 10 weeks of gestation by which time *H19* expression is mostly restricted to the maternal allele [68,69]. DNA methylation of exon 1 of *H19* in placenta shows a significant increase across gestation, from the first trimester to term [70]. Interestingly, there is a significant increase in DNA methylation at a single CpG site within exon 1 of *H19* in PE placentas compared to term (first trimester placentas show no methylation at this site) [70]. The hypermethylation of this single CpG site has been shown to correlate with an increase in proliferation in the human choriocarcinoma JEG-3 cell line [70]. In direct contrast, the methylation of the *H19* differentially methylated region (DMR) is lower at three specific CpG sites in placentas from pregnancies complicated by gestational diabetes mellitus [71].

Aside from the altered *H19* expression observed in placentas from complicated pregnancies, *H19* is down-regulated when a hydatiform mole transitions to choriocarcinoma [72]. The expression of *H19* may inhibit malignant transformation of trophoblast cells into cancer cells, as is supported by the fact that overexpression of *H19* in trophoblast cells leads to reductions in proliferation, migration and invasion [73]. Trophoblast cells, when cultured in the presence of 5-aza-2′-deoxycytidine (a DNA methylation inhibitor), show marked demethylation of a specific CpG site within exon 1 of *H19* (the same CpG site, discussed above, that is hypermethylated in PE placentas), followed by an increase in expression of *H19* [70]. This indicates that the regulation of this one particular site may be important for the expression of *H19*. It should be noted that 5-aza-2′-deoxycytidine acts globally upon the genome, therefore the increased *H19* expression may be a result of reduced DNA methylation elsewhere in the genome.

*H19* expression is restricted to intermediate and villous cytotrophoblasts, and is not found within syncytiotrophoblasts in the human placenta. This expression pattern is consistent with the idea that *H19* helps regulate the invasive properties of intermediate trophoblasts [72,74,75]. DNA methylation analysis of the promotor region of *H19* in placentas from EOPE and PE have demonstrated that the *H19* promoter is hypermethylated in EOPE placentas compared to controls, while the promoter region in PE placentas is not [26,76]. *H19* expression is also significantly lower in EOPE placentas, which is consistent with hypermethylation of the promoter [26,76]. While not statistically significant, DNA methylation of *H19* showed an increase in PE placentas while *H19* expression in PE placentas was lower than control placentas [76]. It is possible that the level of *H19* expression and DNA methylation is linked to the severity and stage of onset of PE observed.

Altered *H19* and *IGF2* expression have been linked to foetal growth restriction and small for gestational age (SGA) in humans [26,77]. A partial loss of imprinting (LOI), together with biallelic expression of *H19*, were detected in placentas from SGA and PE pregnancies [26,73,76]. Similarly, LOI of *H19* and biallelic expression were also linked to foetal growth restriction (FGR) [33] while the maternally inherited single nucleotide polymorphism rs2071094 in *H19* has also been shown to associate with increased birth weight [32]. This LOI may result in an increase in expression of *H19* and mir-675 (a growth suppressor) resulting in SGA and FGR pregnancies. Both a significant increase in *H19* and decrease in *IGF2* expression have been observed in placentas from pregnancies with assisted conception by intracytoplasmic sperm injection (ICSI) and in vitro fertilization (IVF) [78]. This association indirectly provides evidence that there may be a loss of imprinting at the *H19/IGF2* locus in placentas from assisted reproductive technology (ART) pregnancies [78,79] which a meta-analysis has recently shown are more likely to be affected by a wide range of pregnancy complications [80]. Currently there are conflicting reports as to the effect of altered *H19/IGF2* expression on birth weight, with four studies finding an association [26,77,81,82], while three different studies did not observe this association with placentas from ART pregnancies [78,79,83]. Increased 5-hydroxymethlycytosine (5hmC) methylation within the *H19* gene body is positively associated with birth weight, but there is no such association between 5hmC methylation and *H19* gene expression [84].

Together evidence indicates that *H19* plays an important role in placental development. Changes to the DNA methylation status of either the promoter or the gene body of *H19* have been linked with the development of pregnancy complications, PE, EOPE, and IUGR, as well as playing an important role in regulating foetal growth [26,33,77]. This indicates the expression of *H19* is tightly controlled and deviations from the optimal level of expression have detrimental effects on placental function.

## 5. lncRNAs Are Important for Trophoblast Cell Function

HTR8/SVneo is a cell line derived from first trimester extravillous cytotrophoblast cells and is often used for in vitro cell culture experiments. siRNA knockdown of the lncRNA *SPRY4*-*IT1* in these cells leads to an increase in their migratory phenotype, as well as a reduction in the number of dead cells [23]. HTR8/SVneo cells can be cultured on Matrigel to stimulate endothelial-like capillary networks and are a useful cell culture model for studying spiral artery remodelling [85,86]. siRNA knockdown of *SPRY4-IT1* in HTR8/SVneo trophoblast cells leads to a reduced ability of the cells to form tube like networks reminiscent of endothelial tubes, while overexpression of *SPRY4-IT1* leads to an increase in their capacity to form these networks [23]. The mechanism of action of *SPRY4-IT1* in endothelial cell tube-like network remodelling remains unknown, but the expression of *VEGF*, *ANG1* and *ANG2* remain unchanged, indicating it is not via a classical angiogenic pathway [23]. Conversely, when *SPRY14-IT1* is overexpressed (expression plasmid) in HTR8/SVneo cells they show a large reduction in migration [23]. A similar but not significant trend was observed in proliferation assays [23]. Knockdown of *SPRY14-IT1* leads to reduced mRNA and protein expression of E-cadherin and β-catenin mRNA, and an increase in vimentin [87]. *SPRY14-IT1* was shown to bind the cytoplasmic RNA binding protein HuR in HTR8/SVneo cells and the complex formed was then demonstrated to directly bind β-catenin mRNA [87]. The interaction of HuR and β-catenin mRNA has been shown to destabilize β-catenin mRNA in other cell types [88,89]. *WNT3* and *WNT5B* are two downstream targets of β-catenin that are also downregulated as a result of *SPRY14-IT1* knockdown. Tight regulation of Wnt/β-catenin signalling is important for maintaining an epithelial phenotype and cell–cell junctions, and loss of this pathway leads to epithelial-mesenchymal transition (EMT). Extravillous cytotrophoblast cells undergo EMT which is the critical first step for their invasion of the maternal decidua, remodelling of the spiral arterioles and successful pregnancy [90,91].

As mentioned above, *MALAT1* shows altered expression in two pregnancy complications. The function of *MALAT1* was investigated in JEG-3 choriocarcinoma cells and following the knockdown of *MALAT1* expression there was reduced proliferation, migration and invasion and increased cell death [24,29]. Additionally, lower *MALAT1* expression resulted in cell cycle arrest in the G_0_/G_1_ phase [24]. The downregulation of *MALAT1* in HeLa cells also leads to a cell cycle arrest phenotype, but these cells arrested at the G_2_/M phase [92]. *MALAT1* has been shown to act as a molecular sponge (a mechanism by which lncRNAs bind miRNAs preventing them from binding their targets) for miRNAs belonging to the miR-200 family in clear cell kidney carcinoma [93]. The miR-200 family targets *ZEB2*, a transcription factor known for its role in promoting EMT [94,95]; a reduction in *MALAT1* expression can lead to an increase in free miRNAs that can target *ZEB2*. This reduction in *ZEB2* could be responsible for a loss of EMT-like properties observed in JEG-3 cells when *MALAT1* expression is lost.

*MIR503HG* and *LINC00629* are two long intergenic non-coding RNAs (lincRNA) found on the X chromosome between *HPRT1* and *PLAC1*. Expression of *MIR503HG* is similar to that of *PLAC1* with expression found in placenta. Unlike *PLAC1*, *MIRG503HG* expression is also found in human umbilical vein epithelial cells (HUVECS) [96]. By contrast, *LINC00629* is expressed not only in the placenta but also in other tissues such as the ovary, cervix, testis and heart [96]. *MIR503HG* is localised to the nucleus of JEG-3 cells, while *LINC00629* is found evenly throughout the cell. Overexpression of either of these lincRNAs in JEG-3 cells has been shown to reduce both migration (30% lower) and invasion (40% lower) [96]. By contrast, it has been reported that silencing of endogenous *MIR503HG* in HUVEC cells leads to a reduction in cell migration [97]. This evidence surrounding *MIR503HG* function suggests that it may be cell context specific.

**Figure 2:**
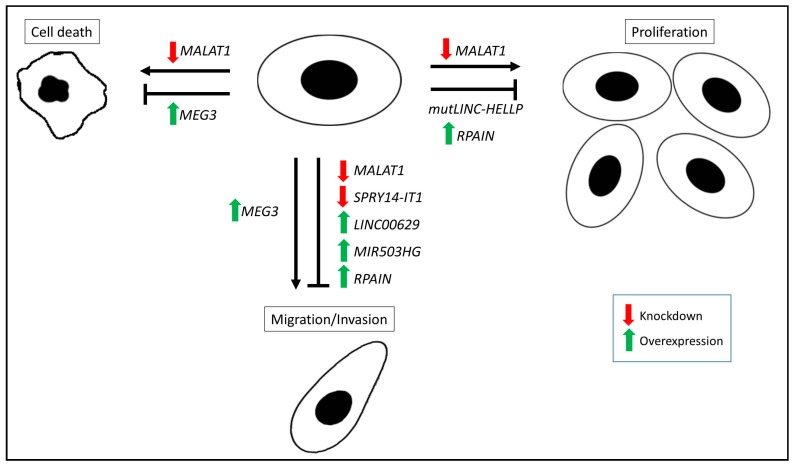
Summary of the roles and function of lncRNAs in trophoblast cells. *MALAT1* and *MEG3* expression influence trophoblast cell death while *MALAT1*, *RPAIN* and *mutLINC-HELLP* influence trophoblast proliferation. Altered *MALAT1*, *SPRY14-IT1*, *LINC00629*, *MIR503HG*, *RPAIN* and *MEG3* expression is associated with changes in trophoblast migration and invasion. Green arrows indicate overexpression of the lncRNA and the impact on cell death, proliferation or migration/invasion while red arrows denote knockdown of lncRNAs and resultant changes in trophoblast function as measured by cell death, proliferation or migration/invasion assays. Black arrows indicate altered gene expression promotes/increases trophoblast function. Black T-bar indicates altered gene expression reduces trophoblast function.

*MEG3* is an imprinted, maternally expressed, lncRNA that has been shown by qPCR to be down-regulated in placentas from preeclamptic pregnancies when compared to normotensive pregnancies [98]. Using the HTR8/SVneo and JEG-3 cells, overexpression of *MEG3* resulted in a reduction in apoptosis and an increase in migration (Figure 2) [98]. A recent study has also shown that *MEG3’s* role in apoptosis and migration may be critical factor in regulating vascular smooth muscle cells during spiral artery remodelling [99]. Another lncRNA identified as altered in preeclampsia is *RPAIN*, which by qPCR was shown to be overexpressed in placentas from preeclamptic pregnancies when compared to uncomplicated controls [100]. Overexpression of *RPAIN* in HTR8/SVneo cells resulted in inhibition of proliferation and invasion. The authors went on to show that C1q, a protein that is important for trophoblast invasion and spiral artery remodelling, was also inhibited when *RPAIN* was overexpressed [100].

lncRNAs are important for a number of critical trophoblast cell functions, from proliferation, invasion and migration, to cell cycle progression. In particular, the altered expression of any of the lncRNAs described above can lead to changes in these functions, and potentially lead to pregnancy complications. So far only a small number of lncRNAs have been studied with respect to their importance in trophoblast cell function, yet a majority influence proliferation and migration, two key processes in trophoblast cell function (Figure 2). It is possible that the strong phenotypes observed upon altering the expression of single lncRNAs are due to their being involved in multiple pathways. Additionally, this emphasizes how complicated molecular control of trophoblast function is, and how delicate the system is, where altered expression of a single lncRNA can have significant downstream effects. Due to knowledge of lncRNA expression and function being in its infancy, it is likely that there are many more unidentified lncRNAs required for trophoblast function, potentially opening up a large future array of therapeutic targets.

## 6. Links between lncRNAs and the Placental Immune/Inflammatory Functions

The placenta is formed from the extraembryonic tissue derived from the conceptus and as such is a unique combination of both the maternal and paternal genomes. Hence, the placenta and the foetus are foreign to the maternal immune system. The development and maintenance of immune tolerance of foetal/placental antigens is a complicated and dynamic process that changes over the course of pregnancy, making it a unique immune state [101]. It includes dramatic changes in the immune profiles in both the maternal circulation and decidua (uterine mucosa in pregnancy) particularly uterine natural killer cells [102], T regulatory cells [103] and M2 macrophages [104]. Since immune tolerance involves changes to both adaptive and innate immunity, pregnant women are at greater risk of infection, especially viral illness. During pregnancy there are several viral pneumonias that have the potential to become more severe than in non-pregnant women, these include but are not limited to influenza, coronavirus, and varicella [105]. Transmission of viral pathogens from the mother to the conceptus can lead to complications in the developing foetus [106].

*lncRHOFX1* is a newly-discovered 4.8-kb lncRNA located on the X chromosome and is one of the most abundantly expressed lncRNAs in the trophectoderm and primitive endoderm [107]. In addition, *lncRHOFX1* is expressed highly within syncytio- and extravillous cytotrophoblast (EVTs) cells obtained by differentiating human embryonic stem cells (hESCs) in an in vitro culture model [108,109,110]. *lncRHOFX1* is also expressed by JEG-3 cells and primary isolated EVTs. *lncRHOFX1* localises to nuclear paraspeckles but it is not known if these speckles are also positive for *NEAT1*.

Knockdown of *lncRHOFX1* in hESC-derived placental cells leads to the increased expression of a number of different immune response genes (Table 2). Trophoblast cells with *lncRHOFX1* knockdown that are subsequently exposed to Sendai virus show reduced expression of viral mRNA compared to control trophoblast cells. This indicates that *lncRHOFX1* expression suppresses antiviral activity. It is proposed that *lncRHOFX1* acts in *trans* to supress the cellular viral immune response [107]. In hESCs, overexpression of *lncRHOFX1* leads to a switch from cell growth to cell differentiation, while no change to antiviral genes is observed [107]. This is another example of cell context influencing the function of lncRNAs.

Further understanding of the molecular regulation of immune tolerance in pregnancy would facilitate better prevention and treatment of pregnancy complications in which it is implicated, as well as new insights into greater prevalence and severity of viral infections in pregnant women.

## 7. Conclusions

In recent years, the number of lncRNAs identified in the human placenta has increased dramatically, and as more RNA sequencing data becomes available, this number is likely to increase. lncRNAs are involved in many different cellular and gene regulatory networks, a discovery that has changed the fundamental understanding of how these networks function and interact with one another. Currently, the specific mechanisms of action for most lncRNAs remain largely unknown, partially due to a lack of conservation across evolution that has limited the suitability of using animal models. This is compounded by the cell type-specific function that a number of lncRNAs exhibit (such as *MALAT1* and *MIR503HG*). Therefore, it is likely that each lncRNA will need to be independently investigated in all cell types in which they are expressed.

Here we have discussed the current literature surrounding lncRNA expression and function in the placenta, trophoblast cells, and how altered expression of several lncRNAs is linked to a number of pregnancy complications. Increasing our understanding of lncRNA function in normal placental development will be critical to unravelling the complex pathogenesis of pregnancy complications and may lead to new diagnostic or therapeutic options.

## Figures and Tables

**Table 1:** Summary of long non-coding RNAs (lncRNAs) linked to pregnancy complications and their functions.

Pregnancy Complication	lncRNA	Status/Function	References
PE	*LOC391533*	Upregulated	[22]
	*LOC284100*	Upregulated	[22]
	*CEACAMP8*	Upregulated	[22]
	*SPRY4-IT1*	Upregulated, increased trophoblast network formation	[23]
	*MALAT1*	Downregulated, decreased trophoblast invasion	[24]
EOPE	*RP11-465L10.10*	Downregulated	[25]
	*H19*	Downregulated, hypermethylated promoter	[26]
HELLP	*LINC-HELLP*	Compound mutations, increase in proliferation and a decrease in both invasion and differentiation	[27,28]
Placenta increta/percreta	*MALAT1*	Upregulated, increase trophoblast invasion	[29]
IUGR	*NEAT1*	Upregulated, disrupts formation of paraspeckles	[30]
	*H19*	Downregulated, hypermethylated promoter	[31,32,33]

PE, preeclampsia; EOPE, early onset preeclampsia; HELLP, haemolysis elevated liver enzymes low platelets syndrome; IUGR, intrauterine growth restriction.

**Table 2:** List of viral sensing genes with altered expression following *lncRHOFX1* knockdown.

Gene Name	Fold Change ^1^	Function
*MX1*	2.24	A guanidine triphosphate metabolizing protein that has antiviral activity against a large number of DNA and RNA viruses [111]. *MX1* is induced by type I and II interferons.
*IFIT1*	2.34	An interferon-induced antiviral RNA binding protein which can selectively bind viral mRNAs and prevent translation [112,113].
*OAS1*	1.95	A double-stranded RNA (dsRNA) binding protein, recognizes and binds dsRNA before recruiting RNaseL [114,115].
*LGALS16*	2.00	A galectin protein found specifically within the syncytiotrophoblast [116] that induces T cell apoptosis as a mechanism of immune tolerance [117].
*IFIH1*	2.23	Both *IFIH1* and *DDX58* are RNA helicases that sense viral RNAs [118]. Both proteins have caspase recruitment domains which can activate type I interferons [119].
*DDX58*	1.97	

^1^ All fold changes represent an increase in expression in *lncRHOFX1* knockdown cells compared to control.

## Abbreviations

RNA	Ribonucleic acid
DNA	Deoxyribonucleic acid
lncRNA	Long non coding RNA
ncRNA	Non coding RNA
miRNA	Micro RNA
siRNA	Small interfering RNA
piRNA	Piwi-interacting RNA
snoRNA	Small nucleolar RNA
tRNA	Transfer RNA
PE	Preeclampsia
qPCR	Quantitative polymerase chain reaction
mRNA	Messenger RNA
EOPE	Early onset preeclampsia
GO	Gene ontology
IUGR	Intrauterine growth restriction
cDNA	Complimentary DNA
SA	Spontaneous abortion
LOI	Loss of imprinting
ICIS	Intracytoplasmic sperm injection
FGR	Fetal growth restriction
IVF	In vitro fertilization
ART	Assisted reproductive technology
DMR	Differentially methylated region
MET	Mesenchymal-epithelial transition
lincRNA	Long intronic non coding RNA
EVT	Extravillous cytotrophoblast
hESC	Human embryonic stem cells
